# Knockout of *TaCKX2.2* in Wheat Improved Grain Yield by Increasing Grain Number Per Spike and Grain Size Under Field Production Conditions

**DOI:** 10.1111/pce.70676

**Published:** 2026-06-14

**Authors:** Zhenzhen Zhu, Fangyao Sun, Guihua Bai, Yanpeng Ding, Fuping Zhang, Xi He, Zhongfu Ni, Qixin Sun, Zhenqi Su

**Affiliations:** ^1^ Frontiers Science Center for Molecular Design Breeding, State Key Laboratory of High‐Efficiency Production of Wheat‐Maize Double Cropping China Agricultural University Beijing China; ^2^ Xinjiang Key Laboratory of Crop Gene Editing and Germplasm Innovation, Institute of Western Agricultural of CAAS Changji China; ^3^ Agricultural College Yangzhou University Yangzhou P.R. China

## Abstract

Knockouts of cytokinin oxidase‐dehydrogenase (TaCKX2.2) homeologs increased grain number per spike, grain size, grain weight per spike, and final yield without negative impacts on other major agronomic traits, revealing a novel approach to improve grain yield by manipulating the TaCKX2.2 gene family in wheat.

Common wheat (*Triticum aestivum* L.) is a staple cereal crop that serves as the primary food source for over half of the global population. Given the limited arable land available and climate change, enhancing wheat yield is imperative to meet the food demands of a growing global population (Shiferaw et al. 2013). Phytohormones, or plant hormones, are involved in various aspects of plant growth and development, such as cell division, elongation, and differentiation, as well as organ formation, and play a pivotal role in regulating grain development and yield formation (Waadt 2020). Therefore, manipulating the genes regulating plant hormone metabolisms offers a viable strategy for increasing grain yield in wheat. Among the phytohormones, cytokinins (CK) were first discovered to be involved in cell division. Several studies have demonstrated its positive effect on grain development, particularly on the determination of grain number and size (Ashikari et al. 2005; Li et al. 2018; Zhang et al. 2012).

Cytokinin oxidase‐dehydrogenase (CKX) is a key enzyme to regulate CK levels by degrading CK irreversibly to maintain the homeostasis of phytohormones, thereby influencing wheat development and yield (Ogonowska et al. 2019). We searched *CKX* in the Chinese Spring reference genome RefSeq v2.0 (https://www.wheatgenome.org/), and found 35 *TaCKXs* homologs that were distributed across 15 of the 21 wheat chromosomes (Figure S1a), and phylogenetic analysis classified them into 11 distinct clusters as previously reported (Shoaib et al. 2019). Among those clusters, *TaCKX2.2* cluster is primarily expressed in spike and grain tissues and has been directly associated with grain yield (https://www.wheatgenome.org/). The cluster comprises five homoeologs in wheat: *TraesCS3A02G311100* (*TaCKX2.2.1‐3 A*), *TraesCS3B02G161000* (*TaCKX2.2.1‐3B*), *TraesCS3D02G143500* (*TaCKX2.2.1‐3D*), *TraesCS3D02G143300* (*TaCKX2.2.2‐3D*), *TraesCS3D02G143200* (*TaCKX2.2.3‐3D*), and each harbors three exons and two introns (Figures 1a, S1b). Amino acid sequence analysis revealed that all members in the *TaCKX2.2* family encode proteins with highly conserved flavin adenine dinucleotide (FAD)‐binding and cytokine‐binding domains at the N terminus and C terminus, respectively (Figure S1c). Expression profiling of the genes in the *TaCKX2.2* subfamily by qRT‐PCR demonstrated that they were predominantly expressed in young developing inflorescence and filling grains after anthesis (Figures S1d and S1e).

**Figure 1:**
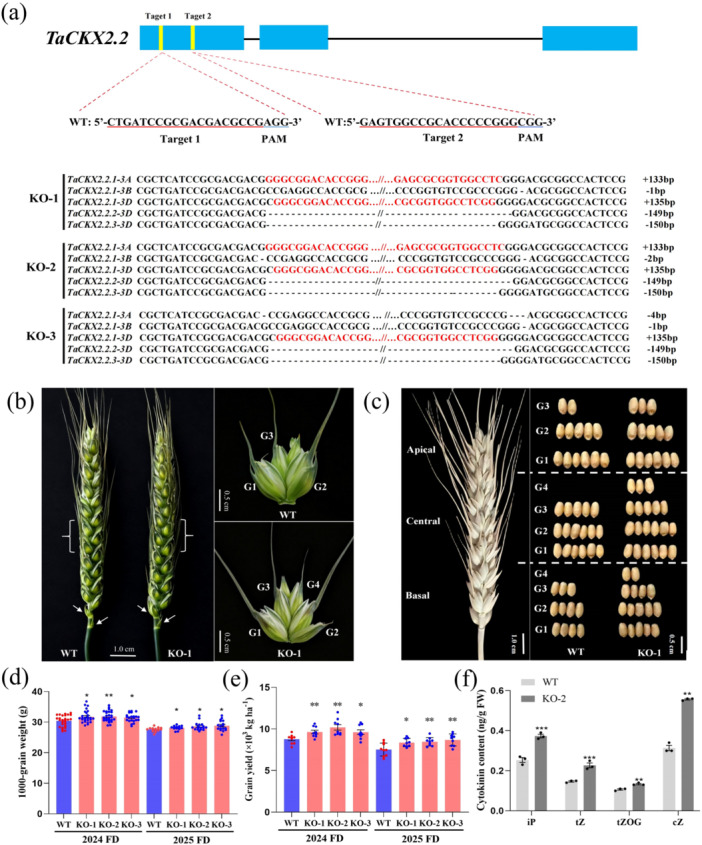
Knocking all *TaCKX2.2* homoeologs out in cv. Fielder using CRISPR/Cas9 significantly increased wheat yield under field conditions. (a) Gene structure of *TaCKX2.2* and edited sequences at the target sites. PAM is protospacer adjacent motifs (underlined in blue), nucleotides labeled with red are inserted sequences, dashes indicate deleted sequences. (b) Representative images of spikes and spikelets of edited lines and wild‐type Fielder (WT). Arrows point to sterile spikelets, G1–G4 denote fertile florets in a central spikelet. (c) Comparison of floret fertility and grain numbers of spikelets from different parts of a spike between a mutant and WT. (d, e) Comparisons of 1000‐grain weight and grain yield between the mutants and the WT. (f) Comparison of various types of cytokinins in young spikes sampled at the jointing‐booting stage between WT and a mutant (n = 3, means ± SE). FW represents fresh weight. Asterisks indicate significant difference between mutants and WT at *p < 0.05, **p < 0.01, ***p < 0.001, and ****p < 0.0001 by Student's two‐tailed *t*‐test. ns = no significant difference. Data are means ± SE.

Wheat is an allohexaploid crop consisting of three sub‐genomes (A, B, D), and each gene usually has at least three homoeologous copies with similar and replaceable functions. To investigate the roles of *TaCKX2.2* members in wheat grain development, two gene‐specific guide RNAs (Figure 1a) were designed to knock out all five homoeologs of *TaCKX2.2* by targeting their coding regions in the cv. Fielder using the CRISPR/Cas9‐mediated gene editing. Sequencing *TaCKX2.2* in the T0 to T4 edited plants identified three mutants (KO‐1, KO‐2, and KO‐3) with edited alleles in all five homoeologs (Figure 1a). In these mutants, the edited alleles of *TaCKX2.2.1‐3 A*, *TaCKX2.2.1‐3B*, and *TaCKX2.2.2‐3D* have frameshift mutations that introduced premature stop codons. *TaCKX2.2.1‐3D* carries an indel complex with a 134‐bp deletion and a 135‐bp insertion that altered the open reading frame, generated a premature stop codon at nt 418–420 downstream of the start codon (ATG), and resulted in a predicted truncated protein. By contrast, the *TaCKX2.2.3‐3D* allele carries a 150‐bp ‐ in‐frame deletion, which removes 50 amino acids from the encoded protein without introducing a premature stop codon. The three selected homozygous lines were subsequently used for further analyses.

In the greenhouses, the *TaCKX2.2* mutant lines showed significant improvements in yield‐related traits compared to their WT control. Specifically, the mutants produced larger grain size (*p* < 0.01), more grains per spike (*p* < 0.01), and fewer unfertile spikelets per spike (*p* < 0.01) than the WT control (Figure S2a–d). To evaluate the impact of the mutants on yield and related agronomic traits under field production conditions, the mutant lines were assessed for those traits in the 2024 and 2025 growing seasons. The results showed that the mutant lines significantly increased grain number per spike (*p* < 0.01), number of fertile spikelets (*p* < 0.01), and grain weight per spike (*p* < 0.01) in the field (Figure S3a‐c). Grain yield of those mutant lines was significantly improved by 9.91% to 13.85% compared to the WT control (Figure 1e), whereas the differences in number of spikelets per spike, tiller number per plant, and plant height were not significant between the WT control and the mutants (Figure S4a–c). We found that the increase in grain number per spike was mainly attributed to an augmented number of fertile florets in a spike, from basal to top spikelets, in all three mutants (WT: 48.35; MU: 54.62) (Figures S3b and 1c). The average 1000‐grain weight of the mutants was higher than the WT control, mainly owing to a significant increase in grain length (Figures 1d and S4d).

Given CKX enzymes negatively regulate CK content (Chen et al. 2020), the content of different CKs in young spikes (~2.0 cm in length) was quantified in both mutant lines and the Fielder control. The spikes were sampled at the jointing‐booting stage. In this stage, wheat florets were forming stamen/pistil, a critical stage to determine floret fertility. The results showed significantly higher content of N^6−^(Δ^2‐^isopentenyl) adenine (iP), *trans*‐zeatin (tZ), *cis*‐zeatin (cZ), and *trans*‐zeatin O‐glucoside (tZOG) in the developing inflorescence of the mutant lines than in the WT control (Figure 1f), indicating that the increase in CK content likely plays a role in enhancing grain number and grain size per spike in the *TaCKX2.2* mutants.

Previous studies have demonstrated that the *TaCKX2.2* subfamily members *TaCKX2.2.1‐3 A* and *TaCKX2.2.1‐3D* negatively regulated grain number and grain size, respectively (Li et al. 2018; Zhang et al. 2012), suggesting the functional differentiation within this gene subfamily. In the current study, we showed that simultaneous mutations of all *TaCKX2.2* homeologues enhanced grain number per spike and grain size, which led to an increase in the grain weight per spike and the final yield, without exerting any negative effects on other major agronomic traits in wheat. This research unveiled a novel approach for developing high‐yield wheat cultivars by manipulating genes in the *TaCKX2.2* family in wheat. Nevertheless, further investigation is needed to elucidate the biological mechanisms of how CK content affects fertile florets in wheat.

## Conflicts of Interest

The authors declare no conflicts of interest.

## Supporting information

Supporting File

## Data Availability

The data that supports the findings of this study are available in the supplementary information of this article.
