# Supplementary material for: Knockout of TaCKX2.2 in Wheat Improved Grain Yield by Increasing Grain Number Per Spike and Grain Size Under Field Production Conditions
Source: Plant Cell Environ. 2026 Jun 14;49(9):6928–30. doi: 10.1111/pce.70676 (PMC13436520; doi:10.1111/pce.70676)
Supplement: Supplementary file 1 [file PCE-49-6928-s001.docx]

**Supplementary Figure S1**. (a) Phylogenetic analysis on *TaCKX* homoeologues in wheat. (b) Comparison of gene structures between *OsCKX2* in rice and *TaCKX2.2* subfamilies in wheat. (c) Amino acid sequence alignment and functional domain analysis of the genes in *TaCKX2.2* subfamily. (d, e) Expression patterns of *TaCKX2.2* subfamily genes in different organs and grains at multiple time points of days post-anthesis (DPA) in cv. Fielder. The transcript levels were determined by qRT-PCR with three replications using Actin (*TraesCS5B02G124100*) as the internal control.

**Supplementary Figure S2.** Comparison of (a) grain number per spike, (b) fertile spikelet number, (c) grain length and (d) grain width between mutant lines and wild type control (WT) Fielder in the greenhouse experiments. Asterisks indicate significant difference between mutant lines and WT at **p* < 0.05 and ****p* < 0.001 by Student’s two-tailed *t*-test, ns refers to no significant difference. Data are means ± SE.

**Supplementary Figure S3.** Comparison of (a) grain number per spike, (b) fertile spikelet number and (c) grain weight per spike between mutant lines and Fielder as the wild-type control (WT) evaluated in field. Asterisks indicate significant difference between mutants and WT at **p* < 0.05, ***p* < 0.01, ****p* < 0.001 and *****p* < 0.0001 by Student’s two-tailed *t*-test. Data are means ± SE.

**Supplementary Figure S4.** Comparison of (a) spikelet number per spike, (b) tiller number, (c) plant height , (d) grain length and (e) grain width between mutant lines and Fielder as wild-type control (WT) in the field production conditions. Asterisks indicate significant difference between the mutants and WT at **p* < 0.05 and ****p* < 0.001 by Student’s two-tailed *t*-test; ns refers to no significant difference. Data are means ± SE.

**Supplementary Table S1.** The primer sequences used for constructing the vector for editing *TaCKX2.2* in Fielder.

| **Primers** | **Sequence (5'-3')** |
| --- | --- |
| MT1T2-F | aataatggtctcAAGCgCTCATCCGCGACGACGCCG |
| MT1T2-F0 | gCTCATCCGCGACGACGCCGgttttagagctagaaatagc |
| MT1T2-R0 | CCCGGGGACGCGGCCACTCCGCTTCTTGGTGCC |
| MT1T2-R | ATTATTGGTCTCTAAACCCCGGGGACGCGGCCACTC |

**Supplementary Table S2.** The subgenome-specific primer sequences used for assays of edited sites in *TaCKX2.2*.

| **Gene IDs** | **Forward sequence (5'-3')** | **Reverse sequence (5’-3’)** |
| --- | --- | --- |
| *TraesCS3A02G311100* | ACAAAGACACAGAGATCGATCC | TGGTCAAGTAAGGAGTACGC |
| *TraesCS3B02G161000* | TAGCCCACCCTCCGCAAC | TGACGTCCAGTTCTTGGACGC |
| *TraesCS3D02G143500* | CGTCCATCACTGTACGTACGTG | GTCGAATAGGTCGGAGTTCCTCC |
| *TraesCS3D02G143300* | AGCCTCTCTCCATAGGCACC | CGCATTTCCAAGGGCAGGG |
| *TraesCS3D02G143200* | CCTCCTCAAGACCGTTCAAGCA | GCCTCCGGCGTCTATGTAT |

**Supplementary Table S3.** The primer sequences used for qRT-PCR assays.

| **Gene IDs** | **Forward sequence (5'-3')** | **Reverse sequence (5’-3’)** |
| --- | --- | --- |
| *TraesCS3A02G311100* | CGAGGAGGCCGGGATAG | GGACAGGATCGCCTTGGGG |
| *TraesCS3B02G161000* | ACGTCGGCGGTGTTCCCT | CCTCTTCCGATCCACGAACCTGGT |
| *TraesCS3D02G143500* | CCCATCCTCGTCTACCCCAT | TACTCCACGCACCGTATCCT |
| *TraesCS3D02G143300* | CATGAACCGGAACAGGTGGG | CAGGTACTGCACGCATCGTA |
| *TraesCS3D02G143200* | ATCGTGCGTGCAGTATCTACC | CTTTCTCTTCCGCTCCATGA |
| *TaActin* | GGAATCCATGAGACCACCTAC | GACCCAGACAACTCGCAAC |

**Methods and materials**

**Sequence analysis, alignment and phylogenetic analysis**

To identify the ortholog of *OsCKX2* in wheat, the sequence of the *OsCKX2* protein was blasted in the Ensemble Plants database (http://plants.ensembl.org). The amino acid sequences of the *CKX2* domains were aligned using ClustalW (Kumar et al., 2016), and the phylogenetic tree was constructed using the neighbor-joining module in the MEGA 7 software (Kumar et al., 2016). The Poisson model was used to calculate evolutionary distance (Hayat and Higgins, 2014). The phylogeny was tested using the bootstrap method with 1000 replications (Saitou and Nei, 1987).

**CRISPR–Cas9-mediated gene editing**

Two sgRNAs were designed to target the first exon of *TaCKX2.2* in the wheat A, B and D genomes (Table S1) using the software in the E-CRISP Design website (http://www.e-crisp.org/E-CRISP/ designcrispr.html). Reverse complementary sgRNA sequences were synthesized, digested, and inserted into the expression cassette of the pBUE411 vector (Xing et al., 2014). The construct was transformed into the wheat cultivar Fielder via Agrobacterium-mediated (EHA105) transformation, following a previously described protocol (Medvecka and Harwood, 2015). The transgenic plants were confirmed by PCR assays to detect the inserted Cas9, the gRNA cassette, and the Bar fragment. To further analyze the positive transgenic plants, genome-specific primers were designed to amplify the target regions in the A, B and D genomes (Table S2). The PCR products were sequenced to identify the *TaCKX2.2* mutant plants.

**Plant material and phenotype analysis**

The wheat variety Fielder was selected for gene editing, and the pBUE411 vector was used for gene editing using a CRISPR/Cas9 system. Two sgRNAs targeting *TaCKX2.2* were simultaneously assembled into the same pBUE411 CRISPR/Cas9 vector as a dual-target construct and introduced into wheat cv. Fielder via Agrobacterium-mediated transformation. The obtained T_0_ seedlings of knockout plants of the *TaCKX2.2* were grown in the greenhouse at China Agricultural University for genotyping and were advanced for four generations to obtain five homozygous mutant lines in the greenhouse. In each generation, mature seeds were manually harvested and cleaned, and dry seeds were stored at -20 °C for further use.

Three randomly selected homozygous lines (KO-1, KO-2, and KO-3) with knockout at all five *TaCKX2.2* homeologues were used for subsequent evaluation of agronomic traits and yield. The wild-type (WT) control used in this study was the recipient cultivar Fielder to ensure the same genetic background between the mutant lines and WT. These three knockout lines and Fielder were grown at the transgenic nursery at the Shangzhuang Experimental Station of China Agricultural University (Beijing, China). In the field, each 2.0 ×1.0 m plot contained six rows with 90 seeds per row. Each line had nine replicates that were arranged in a completely randomized design. Before field harvest, plant height, spike length, number of fertile spikelets per spike, number of grains per spike, and grain yield per spike were manually measured from the main stems in all nine replicates. Thousand-kernel weight, grain length, and grain width of the main spikes were collected after harvesting by selecting well-filled grains using a camera-assisted phenotypic analysis system (Hangzhou Wanshen Detection Technology Co., Ltd., Hangzhou, China).

Final yields in the field trials were evaluated using a method described by Song et al. (2024). In brief, we randomly selected a 1 m² area within each replicate and harvested all the wheat plants in the area. To avoid edge effects, we removed the plants outside the 1 m² area before harvesting.

**Expression pattern analysis**

Gene expression analysis was performed using qRT-PCR. Total RNA was extracted from the several tissues of Fielder, including root, stem, leave, developing inflorescence, and seed at 5, 7, 10, 15, 20 and 25 days after pollination (DAP) using TransZol Plant RNA Kits (TransGen Biotech, ET121-01). All the samples were immediately frozen in liquid nitrogen after collection and stored at -80°C. First-strand cDNAs were generated using the HiScript II Q RT SuperMix (Vazyme Biotech, R223-01) according to the manufacturer’s instructions. Real time-quantitative PCR (RT-qPCR) assays were performed using the SYBR Green PCR Master Mix (Vazyme Biotech, Q121-02/03) in a CFX96 real-time system (Bio-Rad). PCR started at 95°C for 1 min, followed by 45 cycles of 95°C for 10 s, 55°C for 30 s, and 72°C for 1 min, with a final extension at 72°C for 5 min. To standardize the results, the wheat TaActin gene (*TraesCS5B02G124100*) was used as an internal reference for qRT-PCR analysis. The comparative CT (2^-∆CT^) method (Schmittgen and Livak, 2008) was used to quantify gene expression levels based on three biological replicates and three technical replicates for each genes. The primers used in RT-qPCR are listed in Table S3.

**Measurement of endogenous CKs levels**

Developing inflorescences (~2.0 cm in length) were collected from the KO-2 mutant and Fielder at jointing-booting stage, the critical stage for wheat florets to form stamen/pistil and determine floret fertility potential. All samples were immediately frozen in liquid nitrogen after collection and stored at -80°C. Quantification of different types of endogenous CK levels was carried out using the method described by Liu et al. (2010). Briefly, 1 gram of plant tissue powder was extracted using a modified Bieleski solvent (methanol/water/formic acid, 15/4/1, v/v/v), and then isotope-labeled CK compounds were added to the extraction solution as internal quantitative standards. After optimizing PMME with a Shimadzu LCMS-2010EV (Shimadzu, Tokyo, Japan), CK analysis was performed on an Agilent 1200 series HPLC system (Agilent Technologies, Palo Alto, CA, USA). UHPLC-MS/MS analysis was completed by Wuhan Greensword Creation Technology Co., Ltd. (http://www.greenswordcreation.com/index.html). All samples had three biological replicates

**Statistical Analysis**

Statistical analyses on different traits were performed using a two-tailed *t*-test to determine the significance of differences between two groups. “*” indicates a significant difference (*p* < 0.05), “**” indicates a very significant difference (*p* < 0.01), “***” indicates an extremely significant difference (*p* < 0.001), “****” indicates an extremely significant difference (*p* < 0.0001), and “ns” indicates no significant difference (*p* > 0.05). Bar charts for statistical analyses were generated using GraphPad Prism 9.3.1 software.

**SI References**

Feng. Z., Song. L., Song. W., Qi. Z., Yuan. J., Li. R., Han. H. et al. (2021) The decreased expression of *GW2* homologous genes contributed to the increased grain width and thousand‑grain weight in wheat-*Dasypyrum villosum* 6VS·6DL translocation lines. *Theor Appl Genet* 134, 3873-3894.

Hayat, M.J. and Higgins, M. (2014) Understanding Poisson regression. *J. Nurs. Educ* 53, 207–215.

Kumar, S., Stecher, G. and Tamura, K. (2016) MEGA7: molecular evolutionary genetics analysis version 7.0 for bigger datasets. *Mol. Biol. Evol* 33, 1870–1874.

Liu, Z., Wei, F., Feng, Y.Q. (2010) Determination of cytokinins in plant samples by polymer monolith microextraction coupled with hydrophilic interaction chromatography-tandem mass spectrometry. *Anal. Methods* 2 (11), 1676.

Medvecka, E. and Harwood, W.A. (2015) Wheat (*Triticum aestivum L.*) transformation using mature embryos. *Methods Mol. Biol* 1223, 199–209.

Saitou, N. and Nei, M. (1987) The neighbor-joining method: a new method for reconstructing phylogenetic trees. *Mol. Biol. Evol* 4, 406–425.

Schmittgen, T.D. and Livak, K.J. (2008) Analyzing real-time PCR data by the comparative C (T) method. *Nat. Protoc* 3, 1101–1108.

Song, L., Liu, J., Cao, B.L., L, B., Zhang, X.P., Chen, Z.Y., Dong, C.Q. et al. (2023) Reducing brassinosteroid signalling enhances grain yield in semi-dwarf wheat. *Nature* 617, 118–124.

Xing, H.L., Dong, L., Wang, Z.P., Zhang, H.Y., Han, C.Y., Liu, B., Wang, X.C. et al. (2014) A CRISPR/Cas9 toolkit for multiplex genome editing in plants. *BMC Plant Biol* 14, 327.
